# *Runx1* mediates the development of the granular convoluted tubules in the submandibular glands

**DOI:** 10.1371/journal.pone.0184395

**Published:** 2017-09-06

**Authors:** Hitomi Ono Minagi, Safiye Esra Sarper, Hiroshi Kurosaka, Koh-ichi Kuremoto, Ichiro Taniuchi, Takayoshi Sakai, Takashi Yamashiro

**Affiliations:** 1 Department of Oral-facial Disorders, Osaka University Graduate School of Dentistry, Osaka, Japan; 2 Department of Orthodontics and Dentofacial Orthopedics, Osaka University Graduate School of Dentistry, Osaka, Japan; 3 Department of Advanced Prosthodontics, Graduate School of Biomedical & Health Sciences, Hiroshima University, Hiroshima, Japan; 4 Laboratory for Transcriptional Regulation, RIKEN Research Center for Allergy and Immunology, Yokohama, Japan; Rutgers University Newark, UNITED STATES

## Abstract

The mouse granular convoluted tubules (GCTs), which are only located in the submandibular gland (SMG) are known to develop and maintain their structure in an androgen-dependent manner. We previously demonstrated that the GCTs are involuted by the epithelial deletion of core binding factor β (CBFβ), a transcription factor that physically interacts with any of the Runt-related transcription factor (RUNX) proteins (RUNX1, 2 and 3). This result clearly demonstrates that the *Runx /Cbfb* signaling pathway is indispensable in the development of the GCTs. However, it is not clear which of the RUNX proteins plays useful role in the development of the GCTs by activating the *Runx /Cbfb* signaling pathway. Past studies have revealed that the *Runx /Cbfb* signaling pathway plays important roles in various aspects of development and homeostatic events. Moreover, the *Runx* genes have different temporospatial requirements depending on the biological situation. In the present study, the GCTs of the SMG showed a remarkable phenotype of, which phenocopied the epithelial deletion of *Cbfb*, in epithelial-specific *Runx1* conditional knock-out (cKO) mice. The results indicate that *Runx1* works as a partner of *Cbfb* during the development of the GCTs. We also discovered that the depletion of *Runx1* resulted in the reduced secretion of saliva in male mice. Consistent with this finding, one of the water channels, Aquaporin-5 (AQP5) was mislocalized in the cytoplasm of the *Runx1* mutants, suggesting a novel role of *Runx1* in the membrane trafficking of AQP5. In summary, the present findings demonstrated that RUNX1 is essential for the development of the GCTs. Furthermore, RUNX1 could also be involved in the membrane trafficking of the AQP5 protein of the acinar cells in the SMG in order to allow for the proper secretion of saliva.

## Introduction

Various signaling pathways have been shown to play critical roles in the development of the craniofacial organs [1]. The *Runt-related transcription factor (Runx) /core binding factor β (Cbfb)*, signaling pathway plays indispensable roles during bone, tooth, palate and salivary gland development [2]. Various genetic ablation experiments have revealed that each organ and tissue requires a different alpha unit of the RUNX protein; for example, RUNX1 is required for palate development and RUNX2 is required for osteoblast differentiation and tooth development. In our previous study, we ablated *core binding factor β (Cbfb)—*a binding partner of all of the *Runx* genes from the embryonic epithelial tissue, in order to investigate the comprehensive roles of the *Runx /Cbfb* signaling pathway in the development of various tissues. From these studies, we discovered a specific defect in the granular convoluted tubule (GCT), which is an important component of the duct in the submandibular gland (SMG), which developed through the effects on the androgen-dependent gene expression profile. Sexual dimorphism is also evident in the lacrimal glands. Castration-induced testosterone deficiency results in degenerative changes in the lacrimal glands [3]. *Runx1* is expressed in the epithelial compartment of the growing glands and the null mutant observation demonstrates that *Runx1* is involved in gland morphogenesis and in the regulation of regeneration *in vivo* [4]. *Runx1* is also expressed in the epithelium of the prostate glands. The prostate glands are also sexually dimorphic organs and castration results in degenerative changes, indicating androgen-dependent regulation [5]. Although the prostate gland phenotypes have not been reported, a study in which ChIP-seq analysis was performed recently demonstrated that RUNX1 is recruited to androgen receptor binding sites and that Runx1 positively regulates androgen-dependent prostate cancer growth [6].

The mouse GCTs are located between the intercalated and striated ducts, and are larger and more numerous in male glands. The formation of the GCTs occurs in an androgen-dependent manner and develops rapidly in male mice when their cytoplasm testosterone levels increase at the onset of sexual maturity [7, 8]. The GCTs are also reported to synthesize and secrete numerous biologically active polypeptides including epidermal growth factor (EGF), nerve growth factor (NGF), renin, kallikreins and proteases [9]. It is therefore essential for many of the biological activities in the SMG.

We previously showed that the *Runx /Cbfb* signaling pathway plays indispensable roles in the development of the GCTs by genetically ablating *Cbfb* from the developing salivary gland epithelium. However, the specific alpha unit of the RUNX protein that is required in this developmental process remained unclear. We therefore generated epithelial-specific *Runx1* knock-out mice (*Cytokeratin14 (K14) Cre; Runx1*^*fl/fl*^) in order to specify the role of *Runx1* in the development of the GCTs. As we previously reported, in an epithelial *Cbfb* knock-out mouse model, *K14Cre; Runx1*^*fl/fl*^, male mice also showed defective GCT development. Thus, together with our previous report, this result emphasizes the significance of the *Runx /Cbfb* signaling pathway in the GCT epithelium. Moreover it also strongly indicates that *Runx1* functions as a binding partner of *Cbfb*, to drive the *Runx /Cbfb* signaling pathway during the development of the GCTs.

Furthermore, *K14Cre; Runx1*^*fl/fl*^ mice show significantly reduced saliva secretion. In the acinar cells, their water channel (AQP5) was mislocalized. This might be the mechanism underlying their defective saliva production. We herein provide evidence that *Runx1* is the binding partner of the *Cbfb* genes in the SMG and that *Runx1* is involved in sexual dimorphism in the induction of GCTs in the presence of androgen. We also provide dimorphic findings that suggest that RUNX1 could be involved in the membrane trafficking of AQP5 in the SMG.

## Materials and methods

### Animals

All of the animal experiments were performed in strict accordance with the guidelines of the Animal Care and Use Committee of the Osaka University Graduate School of Dentistry, Osaka, Japan. The protocol was approved by the Committee on the Ethics of Animal Experiments of Osaka University Graduate School of Dentistry (permit number: 25-004-0). P50 male and female c57/ BL6 mice were used as wild type (WT) mice. To generate *Runx1* conditional knockout (cKO) mice, we first generated epithelial-specific knockout mice through *the Cre* loxp system to obtain *K14Cre; Runx1*^*fl/fl*^ mice, we then mated heterozygous *K14Cre* mice [10] and homozygous *Runx1*^*fl/fl*^ mice [11, 12]. The progeny were subsequently bred with *Runx1*^*fl/fl*^ mice. Genomic DNA was isolated from tail samples from each mouse using 50 μm NaOH, Tris HCl. Genotyping was performed using a conventional polymerase chain reaction (PCR) method with primer sets to detect *Cre* (5’ CTCTGGTGTAGCTGATGATC 3’ and 5’ TAATCGCCATCTTCCAGCAG 3’) and the loxP site of *Runx1* (5’ CCTCCTCATTCTAACAGGAATC 3’ and 5’ GGTTAGGAGTCATTGTGATCAC 3’). We used their littermates or knockout embryos that did not carry the *K14Cre /Runx1*^*fl/fl*^ genotype as controls. We used *K14Cre; Runx1*^*fl/+*^ and *K14Cre; Runx1*^*+l+*^ mice as controls. All of the study animals were fed powder food.

### Surgery

Male mice were bilaterally orchiectomized (ORX) under ketamine (25 mg /kg) /Rompun (8 mg/kg) anesthesia using sterile conditions. In the male ORX mice, the testes were extracted on P8. The analyses of the ORX mice were repeated at least three times using three mice per group, and the data for a representative experiment were shown.

### The histological analysis

The salivary glands of the mice were dissected on postnatal day 17 (P17) and P50. All of the salivary glands were fixed with 4.0% phosphate-buffered formaldehyde (pH 7.2) and prepared for the histological examination. The sections were stained with hematoxylin and eosin (H&E; Sigma-Aldrich, St. Louis, MO, USA).

### Immunohistochemistry (IHC)

We examined paraffin-embedded salivary gland specimens. The tissue sections were deparaffinized, and antigen retrieval was performed by autoclave heating (instant antigen retrieval H buffer, 121°C for 5 min). The slides were washed in phosphate-buffered saline (PBS). The samples were first incubated with M.O.M. Mice Ig Blocking Reagent (Vector Laboratories, Inc., Burlingame, CA, USA) and then with primary antibodies in diluent (1× PBS, containing 8% protein concentrate; M.O.M.™ Kit; Vector Laboratories, Inc.) overnight at room temperature. The specific antibodies were anti-RUNX1 (dilution 1:500; Sigma-Aldrich, St. Louis, MO, USA), anti-Cytokeratin14 (K14) (dilution 1:100; Everest Biotech, Oxfordshire, UK), anti-E-cadherin (E-cad) (dilution 1:100; BD Biosciences, Franklin Lakes, NJ, USA), anti-Cytokeratin7 (K7) (dilution 1:200; Abcam, Cambridge, England) and anti-Cre (dilution 1:100; CST Japan, Tokyo, Japan). After washing with PBS, the tissues were incubated with Cy2-labelled donkey anti-goat and Cy3-labelled donkey anti-mouse and Cy5-labelled donkey anti-rabbit or anti-rat IgG for 2h at room temperature (dilution 1:100; Life Technologies, Carlsbad, CA, USA) in diluent (5% donkey serum, containing 8% protein concentrate). Immunostaining was repeated at least three times. To quantify the surface area occupied by the ducts, nine representative photos were taken of three SMG sections from independent control mice using an SP8 microscope (Leica, Wetzlar, Germany) under 400× magnification. The images were analyzed using the MetaMorph imaging software program (Leica, Wetzlar, Germany).

### The *in situ* hybridization (ISH) analysis

Digoxigenin-labeled RNA probes were generated for in situ hybridization. Murine *Runx1*, *Crisp3*, *Egf*, *Ngf* probes were synthesized from fragments of *Runx1*, *Crisp3*, *Egf*, *Ngf* (Allen Institute for Brain Science) and were amplified with T7 and SP6 adaptor primers through PCR. We generated 15 μm frozen sections for performing ISH.

### RNA extraction and the real-time RT-PCR

We used Isogen (Nippon gene, Toyama, Japan) to isolate Total RNA from the WT and *Runx1* cKO mice salivary glands on P17 and P50. Reverse transcriptase to cDNA from the total RNA (500 ng) was carried out with an oligo (dT) with avian myeloblastosis virus reverse transcriptase (Takara, Osaka, Japan). The cDNA was used as the template for the quantitative real-time RT-PCR (qPCR). Blend-Taq Plus (Toyobo, Osaka, Japan) was used for the qPCR with a thermal cycler. The sets of synthetic primers that were used for the amplification were as follows: mouse glyceraldehyde-3-phosphate dehydrogenase gene, *Gapdh* (5’ GTCCCGTAGACAAAATGGTG 3’ and 5’ CAATGAAGGGGTCGTTGATG 3’), mouse *Cbfb* (5’ GACAAACACCTAGCCGGGAA 3’ and 5’ GGCTCGCTCCTCATCAAACT 3’), mouse *Runx1* (5’ CTGCAACAAGACCCTGCCCATCGCTTTC 3’ and 5’ CTCCGCCCGACAAACCTGAGGTCGT 3’), mouse *Egf* (5’ TGGAACCCAGTGGAATCCG 3’ and 5’ TGGGATAGCCCAATCCGAGA 3’), mouse *Crisp3* (5’ ACAGTGGCCATTATCCAAGCA 3’ and 5’ GCATGTAGCTAGGCAACGTTTT 3’), mouse *Ngf* (5’ TTTTGATCGGCGTACAGGCA 3’ and 5’ CTGTCACTCGGGCAGCTATT 3’), mouse *Aqp5* (5’ ACCAGATCTCTCTGCTCCGA 3’ and 5’ CCTTGCCTGGTGTTGTGTTG 3’). The qPCR was carried out with *Gapdh* used as a housekeeping gene and the products were analyzed as previously described [13].

### The measurement of the serum testosterone levels

The testosterone levels were measured using an EIA Kit (Cayman Chemical, Michigan USA). The test was repeated at least three times.

### The collection of secreted saliva

The mice were weighed and anesthetized with the intraperitoneal administration of pentobarbital (24 mg/ kg). To stimulate saliva, pilocarpine (0.1 mg/ kg) was injected intraperitoneally. After the injection of pilocarpine, the saliva secreted into the oral cavity was carefully collected using capillaries (Ringcaps; Hirschmann Laborgera GmbH & Co.KG, Eberstadt, Germany) during one-minute intervals for 30 minutes. The total amount of saliva in the control and *Runx1* cKO mice was normalized according to the body weights of the mice.

### Statistical analyses

The statistical analyses were performed as indicated in the Figure legends and text. The differences among means were evaluated by Student’s two-tailed unpaired *t*-test. P values of < 0.05 and <0.01 were considered to indicate statistical significance.

## Results

### The expression of *Runx1* in the salivary glands

**Fig 1:**
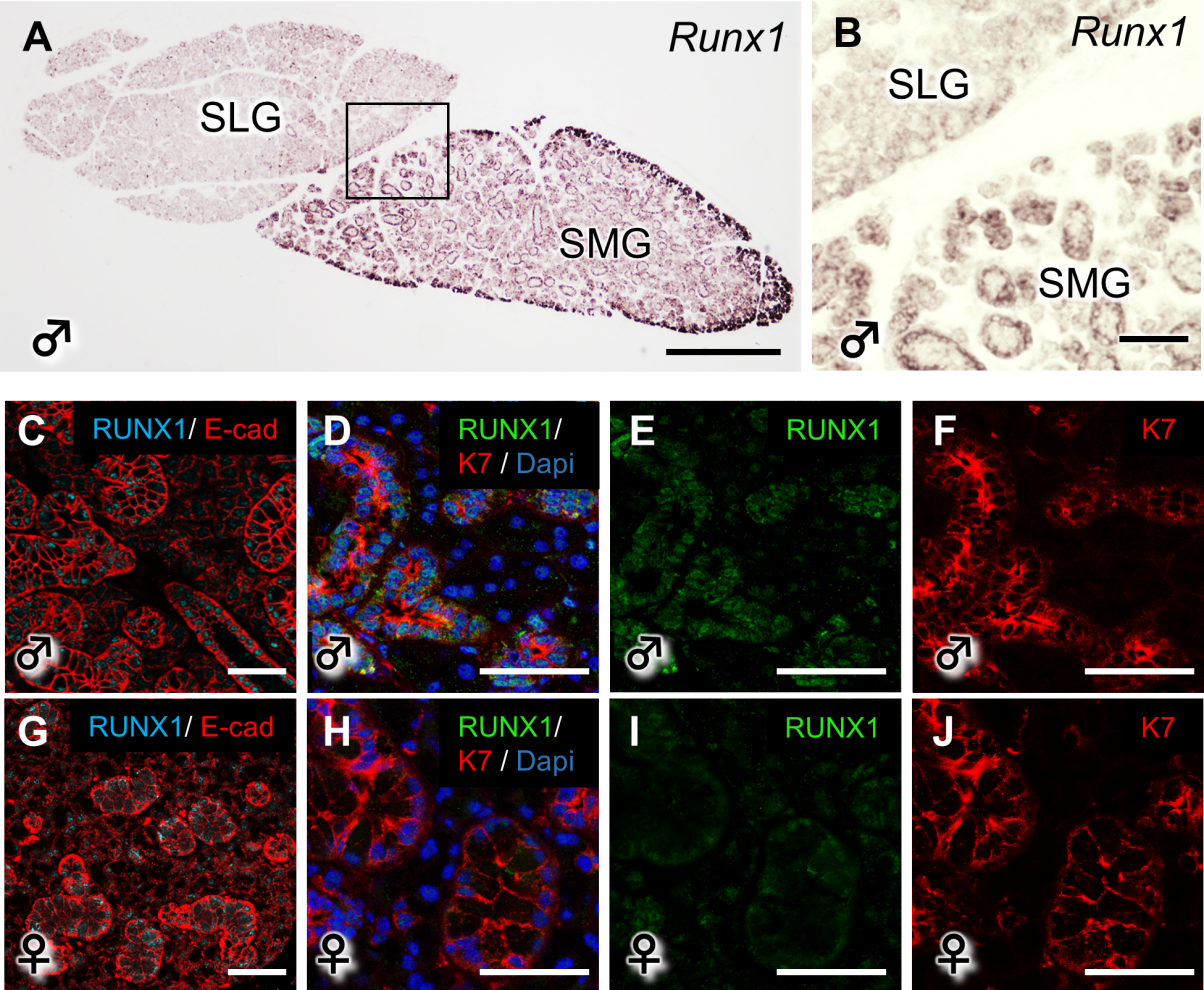
The expression of RUNX1 in the mouse salivary glands. (A, B) The mRNA expression of *Runx1* in the SMG and SLG of P50 WT mice. B shows a higher magnification view of A. Scale bars: 500 μm (A), 100 μm (B). (C-J) The RUNX1 protein expression on P50 in the WT SMG is shown by anti-RUNX1 staining under a confocal microscope. Scale bars: 100 μm.

We evaluated the distribution of *Runx1* mRNA and protein in the SMG and sublingual gland (SLG) of WT mice. The *Runx1* mRNAs were specifically observed in the SMG but not in the SLG (Fig 1A and 1B). In the IHC analysis, which was performed under higher magnification, RUNX1 was found to be most abundantly expressed in the ductal area of the SMG (Fig 1C–1J). E-cad (red), which is a cell adhesion marker, shows the shape of the salivary glands. Moreover, *Runx1* was expressed in the duct area, which was surrounded by K7 a known duct marker[14]. The expression of RUNX1 was showed weak expression in the female SMG. K14 immunoreactivity was broadly observed in the developing ducts during the development of the salivary gland at P17 and P50—especially in the ductal regions (S1A and S1B Fig). Previous reports showed that the expression of K14 appeared to be restricted to the basal excretory ducts and the myoepithelial cells in the adult salivary glands [15, 16].

### The sexually dimorphic GCTs in the male SMG

**Fig 2:**
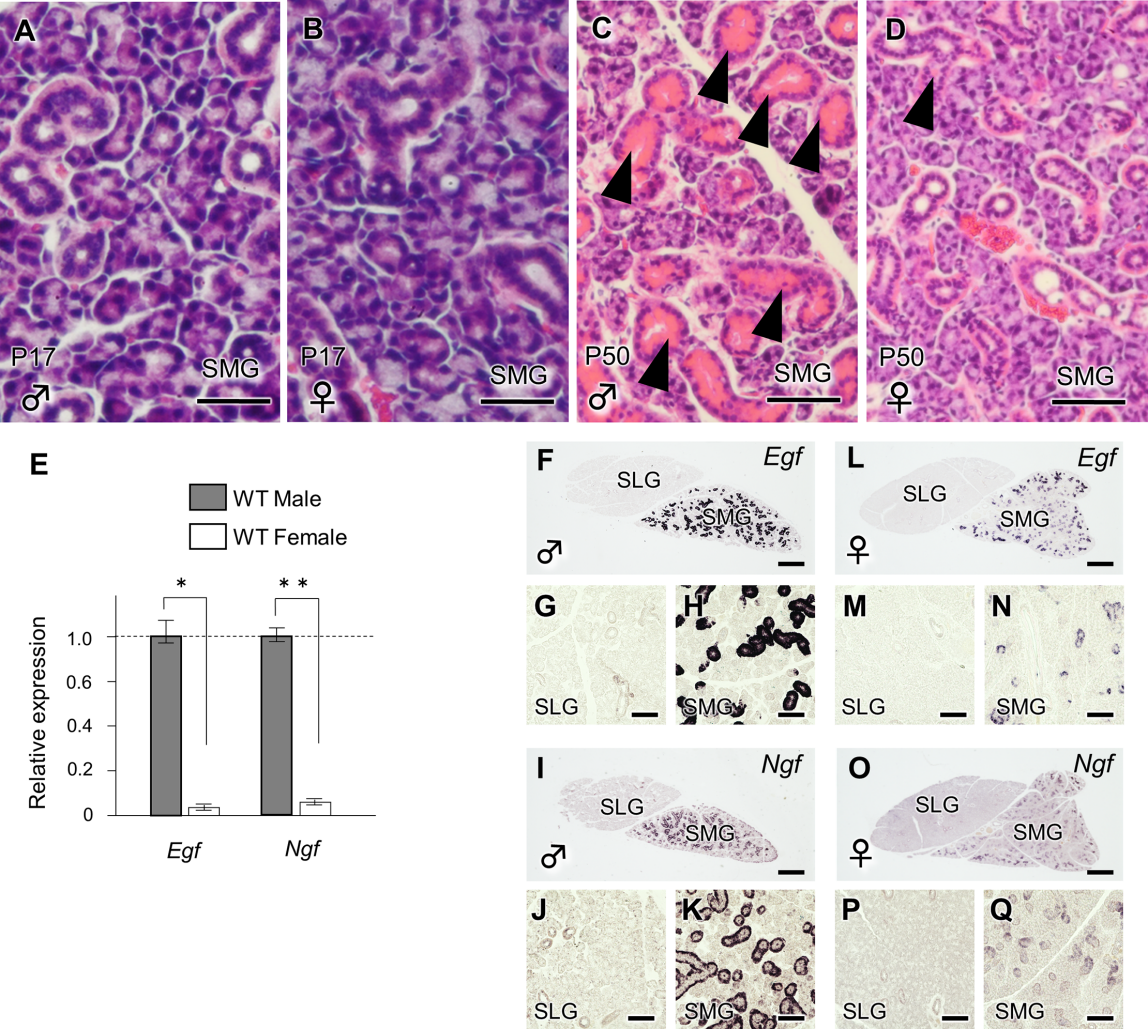
The evaluation of sexual dimorphism in the mouse salivary glands. (A, B) A histological analysis was also performed to compare the male and female WT SMG on P17. Scale bars: 100 μm. (C, D) The histological evaluation of the WT SMG in males and females on P50. The arrowhead indicates the GCT. Scale bars: 100 μm. (E) *Egf* and the *Ngf* mRNA expression in male and female WT mice on P50. The quantification was normalized to GAPDH. *, and ** (F-Q) indicate statistically significant differences (*P*<0.05 and *P*<0.01, respectively) in the mRNA expression of *Egf* and *Ngf* in the WT SMG on P50 males and females. Scale bars: 500 μm. G, H, J, K, M, N, P and Q are higher magnification views of F, I, L and O, respectively. Scale bars: 100 μm.

The GCTs begin to form on P17; thus, sex-related anatomical differences were not observed in the SMG (Fig 2A and 2B). At P50, the GCTs were more prominent in the male SMG (Fig 2C and 2D). Among the SMG, SLG and parotid gland (PG) (all salivary glands), such sexual dimorphism was only evident in the SMG [7]. A qPCR demonstrated that the mRNA expression of *Egf* and *Ngf*, GCT-specific marker genes [17, 18], was significantly higher in the male SMG (Fig 2E). ISH clearly demonstrated that both *Egf* and *Ngf* were specifically expressed in the duct areas of the male SMG, but not of the SLG (Fig 2F–2Q).

### The phenotype of the SMG according to *Runx1* deficiency

**Fig 3:**
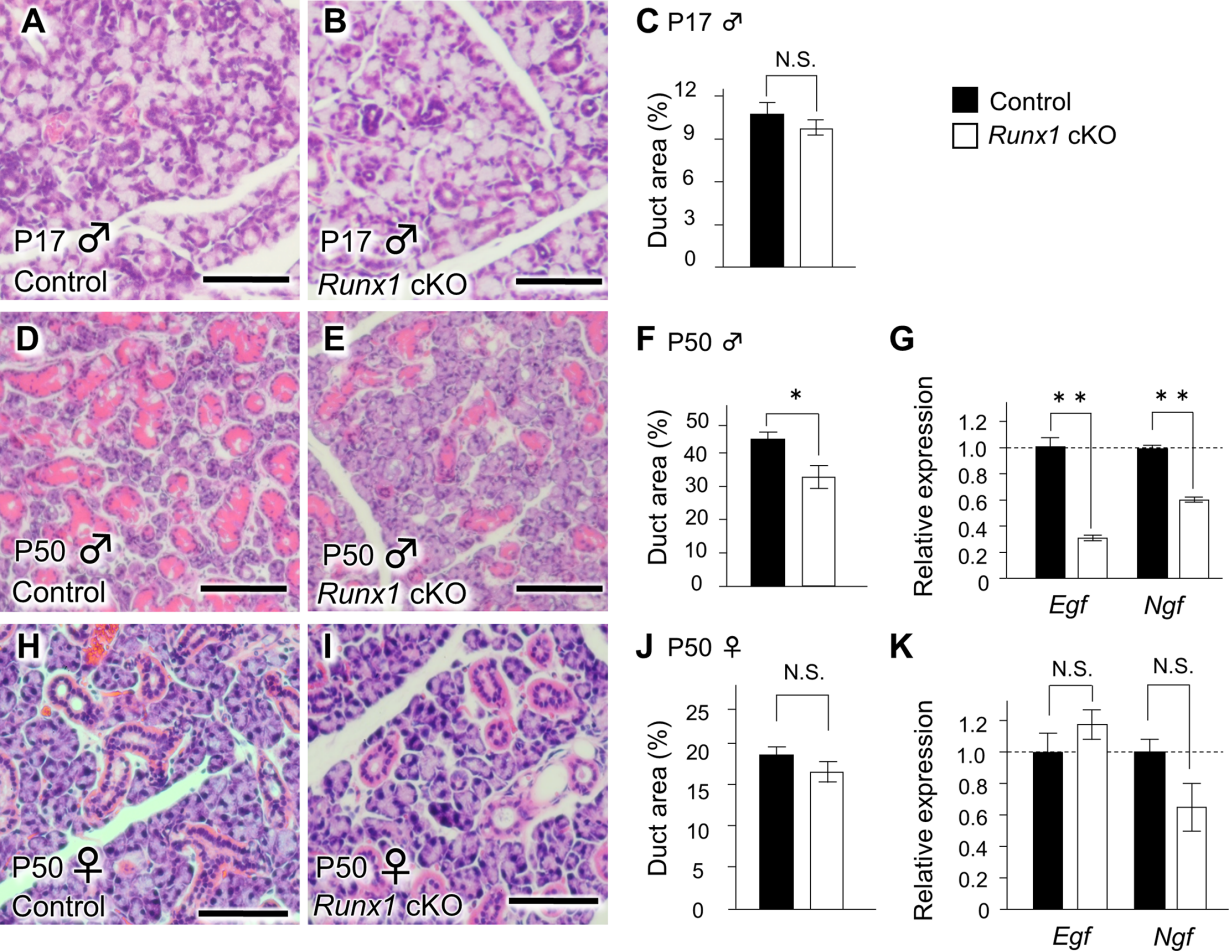
The GCT defect in the male salivary gland of *Runx1* cKO mice on P50. (A, B) The histological appearance of the male SMG in control (A) and *Runx1* (B) cKO mice on P17. Scale bars: 100 μm. (C) A comparison of the duct ratios in the SMG of male control and *Runx1* cKO on P17. (D, E) The histological appearance of the SMG in control (D) and *Runx1* cKO (E) mice on P50 (H&E staining). The *Runx1* GCTs in the SMG of the cKO mice had marked defects in comparison to the control mice. Scale bars: 100 μm. (F) The quantification of the duct ratio in the SMG of male mice on P50. The duct ratio is the duct area per total gland area. *Indicates a statistically significant difference (*P* <0.01) (G) The *Egf* and *Ngf* mRNA expression in the SMG of male mice on P50. **Indicates a statistically significant difference (*P* <0.01) (H, I) H&E staining of SMG specimens from female mice on P50. Scale bars: 25 μm. (J) The duct ratio in the SMG of female mice on P50. (K) The comparison of the *Egf* and *Ngf* mRNA expression in the SMG of female mice on P50. We used their littermates that did not carry th*e K14Cre /Runx1*^*fl/fl*^ genotype as controls.

As shown in Fig 1, at P17, the GCTs were not developed in either the *Runx1* mutant glands or the control glands (Fig 3A–3C). On P50, *Runx1* deficiency did not affect the gross appearance or weight of the male SLG or SMG. However, the GCT defect became significant in the SMG of male *Runx1* mutants at the same stage (Fig 3D–3G). Interestingly, the structure of ducts other than the GCTs, and the acinar cells, were intact in the *Runx1* cKO SMG. A q-PCR confirmed a significant reduction in the mRNA expression of *Egf* and *Ngf* (GCT markers) were significantly reduced by approximately 76.8% and 40.8%, respectively in the SMG of cKO mice (Fig 3G). These findings clearly demonstrate that *Runx1* deficiency resulted in the disordered sexually dimorphic GCTs in the male SMG. This *Runx1* deficiency-inducing chaotic structure of the GCT was not evident in the female glands in either a histological examination (Fig 3H–3J) or a qPCR (Fig 3K).

### Androgen responsiveness and *Runx1* deficiency

**Fig 4:**
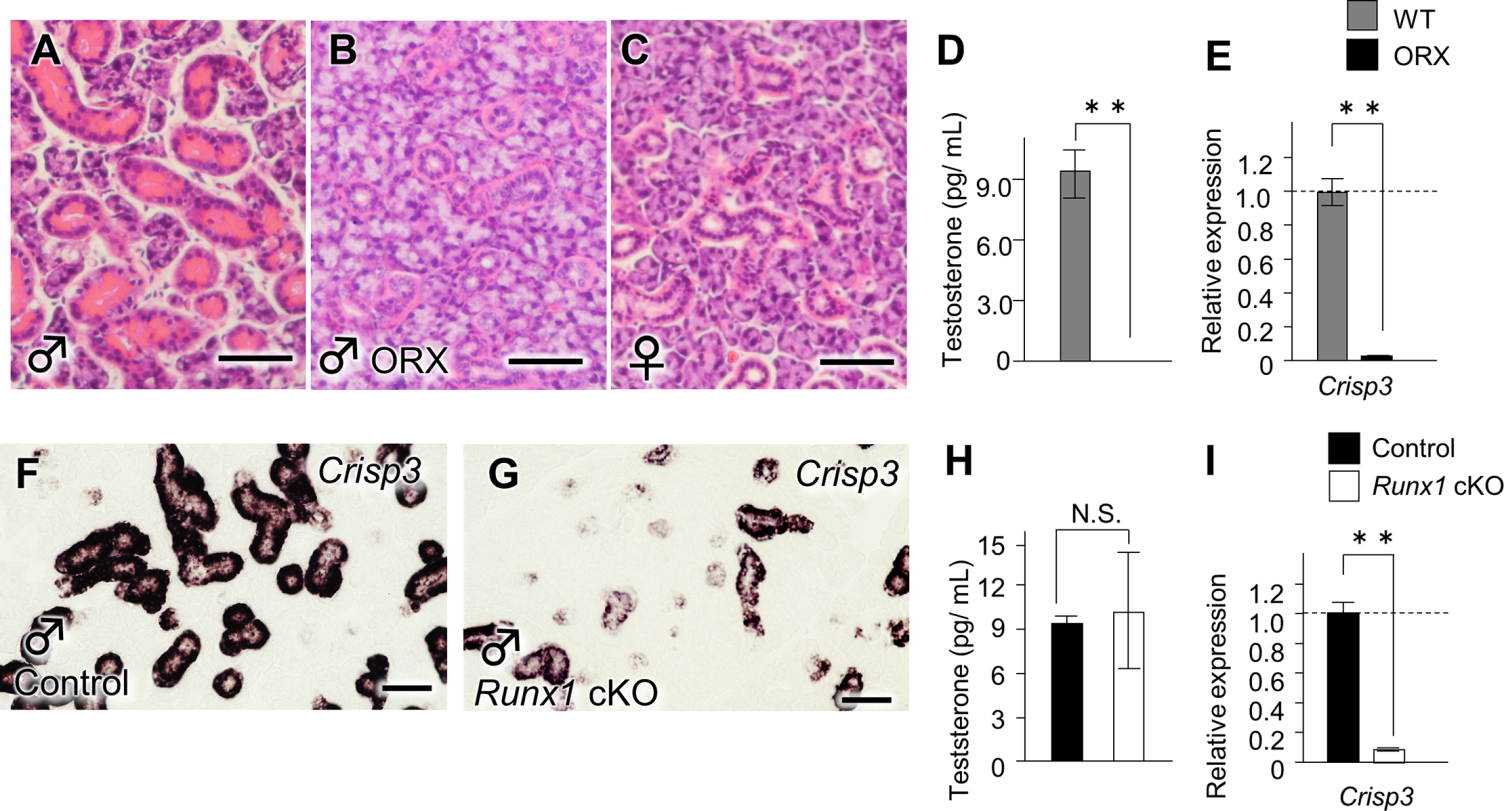
The *Runx1*-androgen receptor pathway. (A) The gross histological appearance of the SMG in WT male mice on P50 (H&E staining). Scale bars: 100 μm. (B) The histological evaluation of the SMG in male ORX mice. The ORX mice, the testes of which were extracted before the development of the secondary sex characteristics, had collapsed GCTs. Scale bars: 100 μm (C) The SMG in WT female mice. Scale bars: 100 μm. (D) The circulating testosterone levels in the male control and ORX mice. **Indicates a statistically significant difference (*P* <0.01) (E) The *Crisp3* mRNA expression in the SMG of control and ORX mice. The gene expression of *Crisp3* was significantly decreased in the SMG of ORX mice in comparison to control mice. (F, G) in the *Crisp3* mRNA expression in the SMG of WT and cKO male mice. Scale bars: 100 μm. (H) The testosterone levels in the serum of control and *Runx1* cKO mice. (I) The *Crisp3* mRNA expression in the SMG of control and cKO male mice.

It is already established that ORX results in the phenotype of the GCTs and leads to diminished levels of circulating testosterone at P50 (Fig 4A–4D) [19]. The GCTs of the ORX mice were similar to those of the controls (Fig 4B and 4C). The ORX mice also showed the considerable downregulation of *Crisp3*, the expression of which is broadly used as a read out of the androgen receptor (AR) pathway [20] (Fig 4E). The present study revealed that *Crisp3* mRNA was distributed in the duct cells of the SMG at p50 (Fig 4F), and the expression profile of which resembles *Runx1* (Fig 1A and 1B). Furthermore, ISH and a qPCR demonstrated that *Runx1* deficiency resulted in the downregulated expression of *Crisp3* (Fig 4F, 4G and 4I). In order to investigate the possibility that the involuted GCTs resulted from the reduced androgen level that occurred through the deletion of RUNX1, the serum testosterone levels, testis phenotypes and serum testosterone levels were evaluated. As a result, we found that *Runx1* deficiency did not affect the testis morphology or the serum testosterone level (4H). Taken together, it is suggested that RUNX1 could directly regulate the sensitivity of the GCTs cells to androgen and contribute to the induction of the GCTs.

### AQP5 in the *Runx1* cKO mice

**Fig 5:**
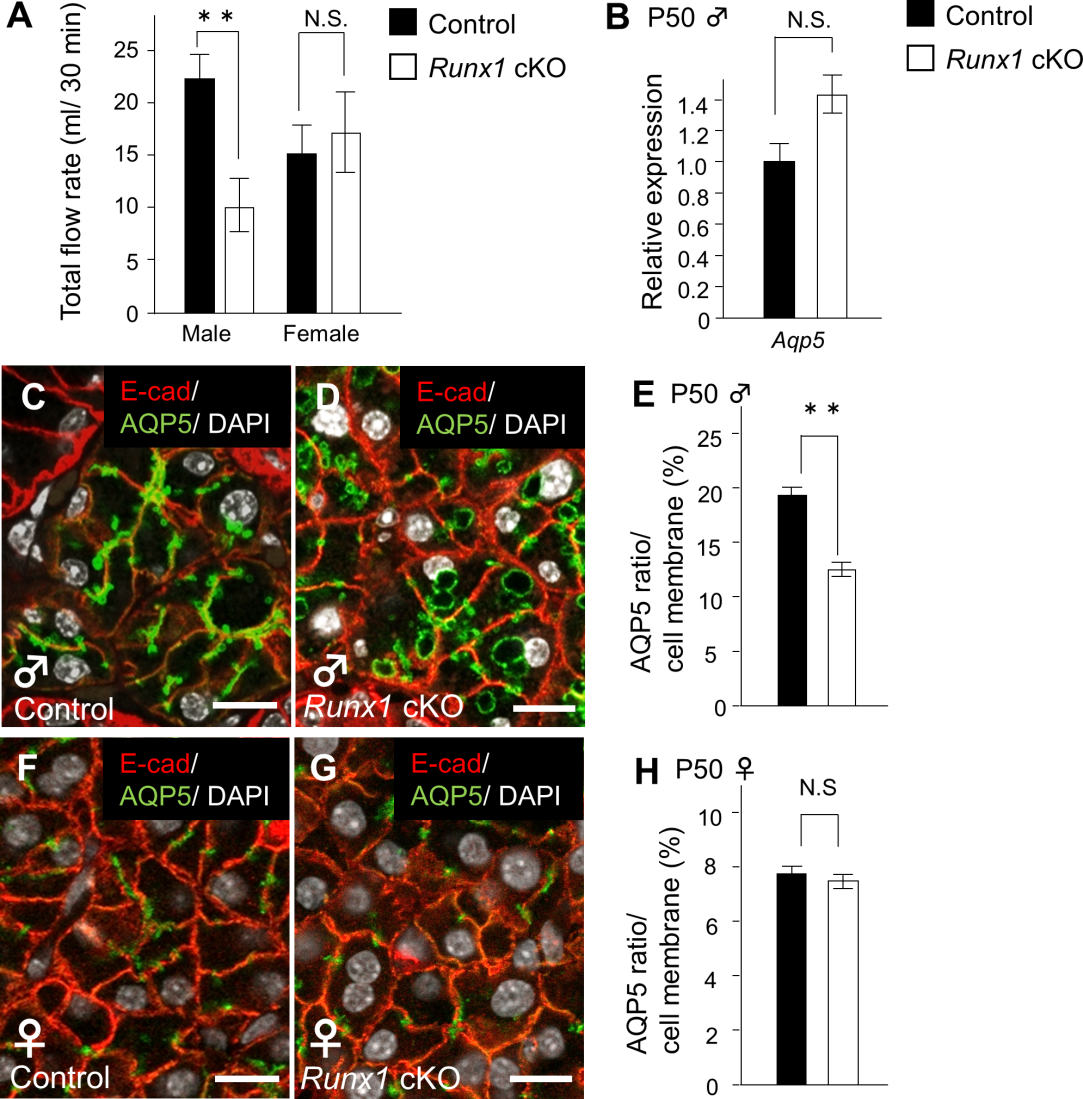
The saliva secretion of the *Runx1* cKO mice. (A) The total volume of saliva secreted in 30 min. (B) **Indicates a statistically significant difference (*P* <0.01) (B) *Aqp5* mRNA expression in the control and cKO P50 male mice as determined by a qPCR. (C, D) The expression of the AQP5 protein in the SMG of male control and *Runx1* cKO mice on P50, as shown by anti-AQP5 staining and confocal microscopy. AQP5 is located in the cytoplasm of the *Runx1* cKO mice. AQP5 (green), E-cad (red), DAPI (white). Scale bars: 50 μm. (E) The AQP5 ratio in the bud cell membranes of control and cKO male mice. The AQP5 ratio represents the localization of AQP5 on the membrane per cell length. (F, G) **Indicates a statistically significant difference (*P* <0.01) in the expression of the AQP5 protein in female mice on P50. There was no difference between the control and the *Runx1* cKO mice. (H) The AQP5 ratio in the bud cell membranes in the female control and cKO mice. We used their littermates that did not carry th*e K14Cre /Runx1*^*fl/fl*^ genotype as controls.

In order to investigate the function of the SMG in *Runx1* cKO mice, we investigated the amount of saliva secreted following an intraperitoneal injection of pilocarpine in comparison to control mice. The saliva production of the *Runx1* mutants decreased to 54.4% at P50. This significant reduction in the secretion of saliva was not observed in the female mutants (Fig 5A). A recent study suggested that the amount of saliva secretion is associated with AQP5—a water channel protein that is specifically present in the salivary glands. In our present study, the *Aqp5* mRNA expression level in the mutant male glands was not affected by *Runx1* deficiency (Fig 5B).

On the other hand, the plasma membrane consists of lipids and proteins, which act as a barrier to water transport, and the trafficking of AQP5 vesicles to the plasma membrane is important in the secretion of water and protein [21]. Hence, AQP5 should be translocated to the apical plasma membrane to secrete saliva into the lumen. Our immunohistochemistry experiments confirmed that AQP5 immunoreactivity was specifically localized along the cell membrane in the glands of WT mice (Fig 5C). In contrast, the AQP5 immunoreactivity of the membrane of the *Runx1* mutants was decreased, while the immunoreactivity of the cytoplasm remained (Fig 5C–5E). On the other hand, there was no evidence to suggest that the trafficking of AQP5 was disturbed in the SMG of female *Runx1* mutants or female WT mice (Fig 5F–5H). Our findings suggested that the AQP5 protein was still present in *Runx1* mutants, but that the trafficking of AQP5 to the cell membrane was disturbed.

## Discussion

The role of the *Runx /Cbfb* signaling pathway has been shown to be critical to many biological aspects including embryonic development and the tissue-specific homeostasis-like maintenance of stem cells [22]. In recent years, this signaling pathway has been analyzed using conditional alleles to investigate its detailed roles in various situations [23]. Our previous study showed that the GCTs showed a chaotic structure after the epithelial deletion of *Cbfb*, which strongly suggests the significance of *Runx /Cbfb* signaling in the induction of the GCTs [24]. *Cbfb* is an essential binding partner of the *Runx1*, *2* and *3* genes, to form heterodimeric core-binding transcription factor [25]. However, the detailed counterpart of CBFβ has not been completely identified in various situations. In the present study, the defective phenotypes of the GCTs of *Runx1* cKO mice were similar to those that occurred after the epithelial deletion of CBFβ, indicating that RUNX1 plays a dominant role in governing the *Runx /Cbfb* signaling pathway in the epithelium of the GCTs.

The disordered polarity of the GCTs in *Runx1* mutants was associated with a decrease in the androgen pathway activity without a corresponding decrease in the androgen level. Indeed the physiological roles of the sexual hormones and their influence on organ morphogenetic differences are largely unknown. A clearer understanding of sexual dimorphism will be useful for the prevention of diseases that are more prevalent in either gender—or for the development of novel treatment strategies for such diseases. The salivary glands have important functions in maintaining oral health [26, 27]. Sex hormones have been suggested to play a role in salivation; however, the underlying molecular mechanisms are currently unclear [28]. The induction of the GCTs is androgen-dependent and the GCTs show sexual dimorphism. The development of GCT dimorphism is induced by increased levels of testosterone at the onset of sexual maturity. The GCT cells have secretory granules (that are specifically abundant), which contain a variety of biologically active peptides [7, 20]. It has been established that the involution of the GCT is induced by a castration-inducing reduction of the androgen levels in male mice [29].

The involution of the GCTs in *Runx1* mutants was associated with a decrease in the androgen pathway activity without a corresponding decrease in the androgen levels. Since the female *Runx1* mutant glands did not show the involution of the GCTs and because the involution in the *Runx1* mutants was not evident at the embryonic stage, RUNX1 could mediate androgen signaling in the presence of serum testosterone in the induction of the GCTs. In other words, sexual dimorphism in the salivary glands is mediated by *Runx1 /Cbfb* signaling via the triggering and/or activation of the androgen-dependent response. Salivary gland sexual dimorphism is specific to the SMG. This specificity is supported by the expression pattern of *Runx1* and *Cbfb*, both of which are specifically expressed in the SMG. Furthermore, the distribution of *Crisp3*, a biomarker of the androgen pathway, is similar to that of *Runx1* and *Cbfb*. This overlapping expression pattern also supports the essential roles of *Runx /Cbfb* signaling in salivary gland sexual dimorphism via the regulation of the androgen pathway. In the present study, we also found that saliva secretion was disturbed by the depletion of *Runx1* and that this salivary phenotype was specific to male mice. AQP5 is a salivary gland-specific water channel, which provides the major route for water secretion across the apical membranes of the acinus [30, 31]. Actually, the production of saliva in *Aqp5* null mutants is reduced by more than 60% [32], and the polarity of AQP5 has been found to be disturbed in patients with Sjögren’s syndrome [33, 34]. It was interesting to find that the mRNA expression of *Aqp5* was not reduced by *Runx1* deletion; however, *Runx1* deficiency significantly affected the cytolocalization of the immunoreactivity to AQP5 in the acini of the SMG. The water channel of AQP5 protein should be distributed in the cell membranes to function as a water channel and secrete water—while the alveolar membrane of *Runx1* mutants shows decreased immunoreactivity and the cytoplasm shows increased immunoreactivity. These findings suggest that decreased levels of testosterone do not induce aberrant membrane trafficking. At present, the mechanism should be elucidated. Our hypothesis is that growth factor signaling disturbs the transportation of proteins. Many of these growth factors may function directly or indirectly in the maintenance and stimulation of the salivary gland tissues [35]. One of the GCT markers, the EGF family, interacts with the stimulated pathways [36]. On the other hand, such phenotypes were not observed in the female *Runx1* mutants, indicating that dimorphism also occurs in the membrane trafficking of AQP5 proteins in the acinus. A clearer understanding of sexual dimorphism will be useful for the preventing diseases of the salivary glands as well and for developing treatment strategies for such diseases. These results might help us to understand the pathogenesis of Sjögren’s syndrome and certain forms of s diseases that affect the salivary glands. Sjögren’s syndrome is autoimmune disease of the salivary and lacrimal glands that is characterized by dry mouth and eyes. Women typically develop this disease at the time of menopause. Sex hormones may be involved in the pathogenesis. However, estrogen deficiency alone does not account for the development of the disease. Dehydroepiandrosterone (DHEA) is produced by the adrenal glands and DHEA transform into androgens and/or estrogen in the peripheral tissues, and women in menopause also develop a lack of androgens due to the marked decrease in adrenal DHEA levels [37]. Indeed, Sjögren’s syndrome patients demonstrate marked decreases in their serum DHEA and androgen levels and reduced androgen receptor activity, as revealed by the decreased expression of CRISP-3 [38, 39]. On the other hand, treatment with testosterone was shown to significantly reduce the inflammation in the salivary glands of a female mouse model of Sjögren’s disease [40], suggesting that androgen protect against degenerative changes in salivary gland tissue. Collectively, androgens is involved in, at least in part, the development and maintenance of the syndrome. The present study clearly showed *Runx1* deficiency *in vivo* impaired the induction of male dominant dimorphism that is accompanied by a disturbed androgen pathway. Although direct association of *Runx1* in pathogenesis of Sjogren syndrome has not been reported, a part of downstream molecular pathway might be shared in development of the disease.

## Conclusions

In summary, the comparison of the phenotypes of the present *Runx1* mutants and the previous *Cbfb* mutants suggested the important roles of *Runx1* in androgen-dependent sexual dimorphism in the SMG. RUNX1 could be involved in sexual dimorphism in induction of the GCTs in the presence of androgen. Moreover, *Runx /Cbfb* signaling could regulate the membrane trafficking of the AQP5 protein in the acinar cells of male mice.

## Supporting information

S1 Fig The identification of K14-expressing cells in the control SMG. (A, B) Immunofluorescence images of SMG sections obtained from male mice that were co-immunostained with antibodies to K14 (blue) and E-cad (red). K14-expressing cells were observed in the ductal basal and myoepithelial cells of the control SMG on P50 and P17. Scale bars: 50 μm. (C-F) Immunofluorescence images of SMG sections obtained from male mice that were co-immunostained with antibodies to K14 (red), Cre (green) and DAPI (blue).
(TIF)

S2 Fig A diagram of the present study. The GCTs in the SMG of male epithelial-specific *Runx1* cKO mice were disrupted and the expression of the androgen-dependent transcript gene was downregulated. The saliva flow rates of male *Runx1* cKO mice were disrupted and the localization of AQP5 showed that the loss of *Runx1* inhibited the membrane trafficking of AQP5. The circulating testosterone levels were not affected in the *Runx1* cKO mice, indicating that the duct phenotypes could be organ-level effects of epithelial *Runx1* deficiency. There were not functional and structural defects occurred at control and cKO female mice. These phenomena occur in an androgen-dependent manner with the development of secondary sex characteristics.
(TIF)

## Abbreviations

AQP: Aquaporin
CBFβ: core binding factor β
cKO: conditional knock-out
DHEA: dehydroepiandrosterone
E-cad: E-cadherin
EGF: epidermal growth factor
GCT: granular convoluted tubule
IHC: immunohistochemistry
ISH: *in situ* hybridization
K: cytokeratin
NGF: nerve growth factor
ORX: orchiectomized
PBS: phosphate-buffered saline
PG: parotid gland
qPCR: real-time RT-PCR
P50: postnatal day
Runx: Runt-related transcription factor
SLG: sublingual grand
SMG: submandibular gland
WT: wild type
ZO-1: zona occludens protein-1
