# Supplementary figures and images for: Runx1 mediates the development of the granular convoluted tubules in the submandibular glands
Source: PLoS One. 2017 Sep 6;12(9):e0184395. doi: 10.1371/journal.pone.0184395 (PMC5587342; doi:10.1371/journal.pone.0184395)

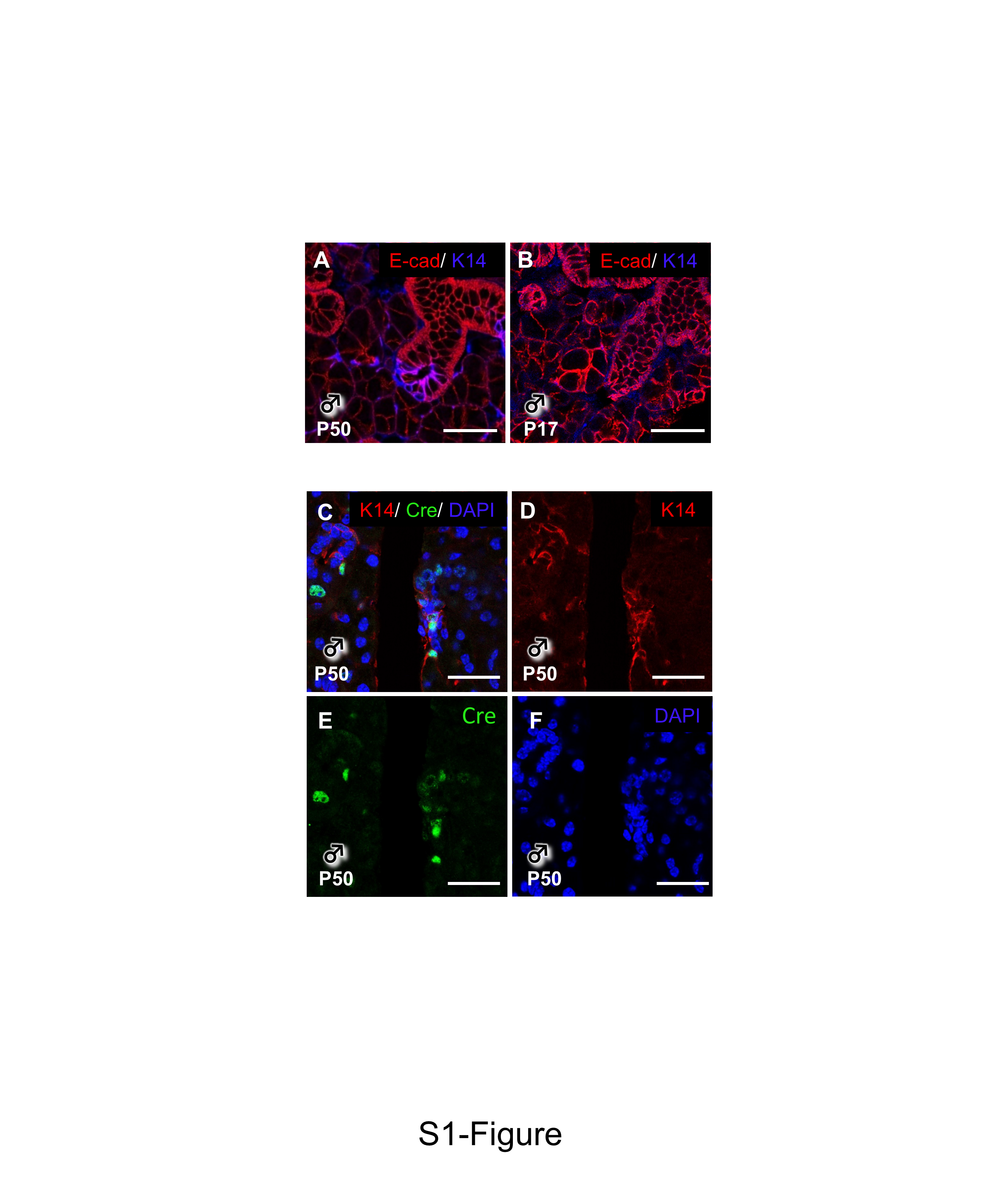

Supplement: S1 Fig — (A, B) Immunofluorescence images of SMG sections obtained from male mice that were co-immunostained with antibodies to K14 (blue) and E-cad (red). K14-expressing cells were observed in the ductal basal and myoepithelial cells of the control SMG on P50 and P17. Scale bars: 50 μm. (C-F) Immunofluorescence images of SMG sections obtained from male mice that were co-immunostained with antibodies to K14 (red), Cre (green) and DAPI (blue). (TIF) [file pone.0184395.s001.TIF]

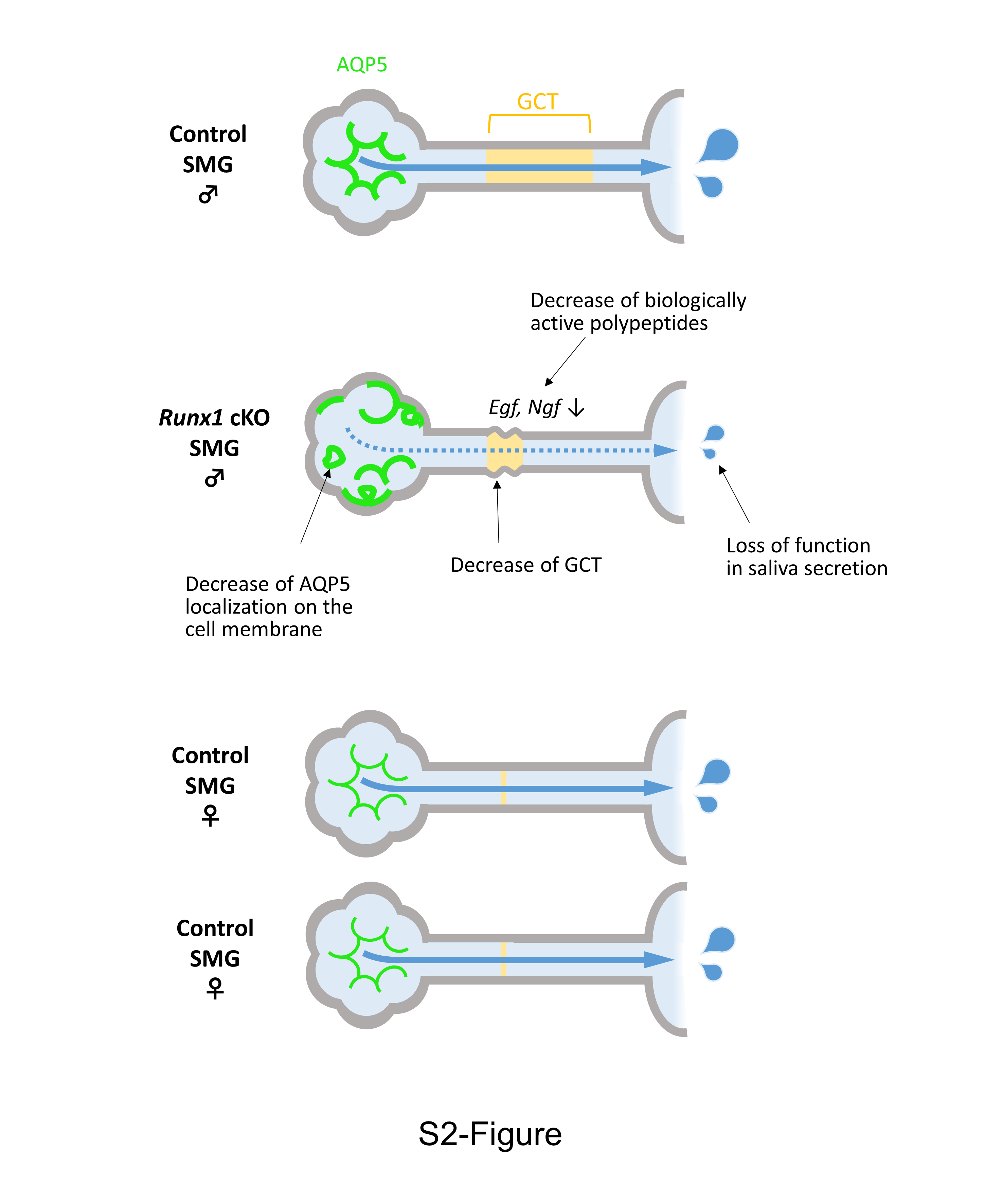

Supplement: S2 Fig — The GCTs in the SMG of male epithelial-specific Runx1 cKO mice were disrupted and the expression of the androgen-dependent transcript gene was downregulated. The saliva flow rates of male Runx1 cKO mice were disrupted and the localization of AQP5 showed that the loss of Runx1 inhibited the membrane trafficking of AQP5. The circulating testosterone levels were not affected in the Runx1 cKO mice, indicating that the duct phenotypes could be organ-level effects of epithelial Runx1 deficiency. There were not functional and structural defects occurred at control and cKO female mice. These phenomena occur in an androgen-dependent manner with the development of secondary sex characteristics. (TIF) [file pone.0184395.s002.TIF]
